# Construction and immunological characterization of CD40L or GM-CSF incorporated Hantaan virus like particle

**DOI:** 10.18632/oncotarget.11329

**Published:** 2016-08-17

**Authors:** Qikang Ying, Tiejun Ma, Linfeng Cheng, Xiaoxiao Zhang, Agnieszka D. Truax, Ruixue Ma, Ziyu Liu, Yingfeng Lei, Liang Zhang, Wei Ye, Fanglin Zhang, Zhikai Xu, Lei Shang, Rongrong Liu, Fang Wang, Xingan Wu

**Affiliations:** ^1^ Department of Microbiology, School of Basic Medicine, Fourth Military Medical University, Xi'an, 710032, China; ^2^ The Lineberger Comprehensive Cancer Center, University of North Carolina at Chapel Hill, Chapel Hill, NC 27599-7295, USA; ^3^ Department of Statistics, Fourth Military Medical University, Xi'an, 710032, China

**Keywords:** Hantaan virus, virus like particle, virus vaccine, CD40 ligand, granulocyte macrophage colony-stimulating factor

## Abstract

Infection of Hantaan virus (HTNV) usually causes hemorrhagic fever with renal syndrome (HFRS). China has the worst epidemic incidence of HFRS as well as high fatality. Inactivated whole virus has been used for HFRS vaccination, however there are still problems such as safety concerns. CD40 ligand (CD40L) and granulocyte macrophage colony-stimulating factor (GM-CSF) are well-known immune stimulating molecules that can enhance antigen presenting, lymphocytes activation and maturation, incorporation of CD40L and GM-CSF to the surface of virus like particles (VLPs) can greatly improve the vaccination effect. We constructed eukaryotic vectors expressing HTNV M segment and S segment, as well as vectors expressing HTNV M segment with CD40L or GM-CSF, our results showed successful production of CD40L or GM-CSF incorporated HTNV VLPs. *In vitro* stimulation with CD40L or GM-CSF anchored HTNV VLP showed enhanced activation of macrophages and DCs. CD40L/GM-CSF incorporated VLP can induce higher level of HTNV specific antibody and neutralizing antibody in mice. Immunized mice splenocytes showed higher ability of secreting IFN-γ and IL-2, as well as enhancing CTL activity. These results suggest CD40L/GM-CSF incorporated VLP can serve as prospective vaccine candidate.

## INTRODUCTION

Infectious Hantaan virus (HTNV) is a major cause of hemorrhagic fever with renal syndrome (HFRS). In China, it refers to about 100,000 of patients every year [1]. Inactivated whole HTNV viron has been used for HFRS vaccination, and the outcome is encouraging [2]. However the inactivated vaccine still has problems with less than satisfactory levels of neutralizing antibody titer and activation of cellular immunity. In addition there are also safety concerns with bringing in animal-derived virus or virulence restoration [3]. Therefore, development of new type of HFRS virus vaccine is imminent for the control of HFRS.

HTNV viral genome contains three fragments, L, M and S, which encode RNA depended RNA polymerase (RdRp), membrane glycoprotein (GP, GP is further cleaved into Gn and Gc) and nucleoprotein (NP) respectively [4]. Both GP and NP are the main immune response eliciting structural proteins. GP is responsible for viral entry [5] and is able to induce neutralizing antibodies that, facilitate protective immune response, but its immunogenicity is relatively weaker [6]. NP has several T cell epitopes and has strong immunogenicity [7], however the ability to induce neutralizing antibodies is lower. Virus like particles (VLPs) that mimic natural virus can provide a way to combine both GP and NP to boost both humoral and cellular immune response.

VLPs usually emerge in natural virus life cycles, which consist of viral structural protein [8]. The formation of VLPs can be achieved by simply expressing one or several factors like structural proteins [9], and can be genetically altered to insert foreign gene and display desired molecular on the surface [10, 11], they are widely used in researches of vaccines and mechanism of virus infection. The VLP approach appears promising and advantageous over many other structural forms of vaccines [12–14]. VLPs have been found to provide high immunogenic potency in protecting against various pathogens, such as human papillomavirus. The FDA approved HPV vaccine Cervarix^®^ and Gardasil^®^ are composed of VLPs which can provide cross protection for different HPV serotypes, also induce ten times higher neutralizing antibodies than natural infection [15, 16]. Although those are the only two approved VLP vaccines nowadays, there are many more in development [17].

It has been mentioned above that the expression of M segment in host cells can lead to production of VLPs that were composed of spherical membrane vesicles, with or without nucleocapsid, similar to the morphology of natural virions, but we need VLPs with full components in order to elicit both neutralizing antibodies and virus-specific cytotoxic T lymphocytes. In order to enhance the immunogenicity, the gene expressing VLPs can be altered to display non-structural proteins such as cytokines and specific antigens [18] that usually won't abrogate the expression of structural protein. CD40L and GM-CSF are usually used to enhance immune effect of vaccines [19]. CD40L is a key activator of immune response by engaging CD40 on the B cell surface and therefore facilitating immunoglobulin class switching and affinity maturation, which is an important process of adaptive immune response [20, 21]. GM-CSF is identified as an inducer of differentiation and proliferation of granulocytes and macrophages derived from hematopoietic progenitor cells, it also involves an adaptive immunity by activation of dendritic cells [22]. Both CD40L and GM-CSF is able to facilitate anti-viral immunity, so we choose the two factors to construct recombinant VLPs.

In this study we constructed plasmids containing HTNV M segment and CD40L/GM-CSF gene, co-transfected with vector containing S segment into *dhfr*^−^ CHO and obtained HTNV VLPs anchoring CD40L or GM-CSF, the protein composition and the structure were verified by Western blot. *In vitro* stimulation with CD40L or GM-CSF anchored HTNV VLP showed enhanced activation of macrophages and DCs. Data of animal research suggest that humoral immune responses and cellular immunity induced by CD40L or GM-CSF decorated HTNV VLP were superior to undecorated VLPs as well as HTNV vaccine, and able to reduce viral load in immunized mice. These results indicated that the recombinant HTNV VLP could be a promising vaccine candidate.

## RESULTS

### CD40L/GM-CSF and HTNV GP can be expressed by co-transfection of pCI-S and pCI-M-CD40L, pCI-S and pCI-M-GM-CSF

**Figure 1:**
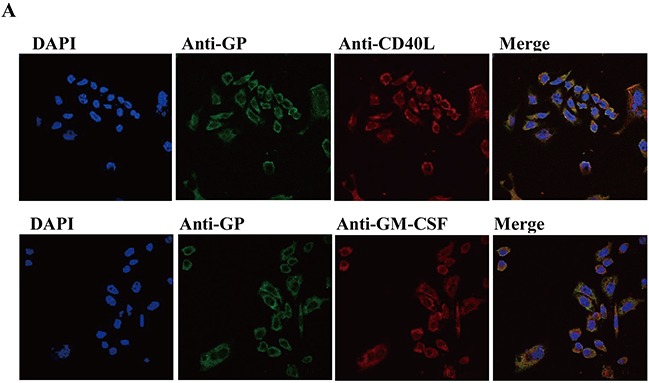
Verification of plasmids Co-transfection of pCI-S and pCI-M-CD40L, pCI-S and pCI-M-GM-CSF into *dhfr* deficient CHO cells and the expression of GP, CD40L and GM-CSF were tested using GP specific monoclonal antibody (mouse) and CD40L/GM-CSF polyclonal antibody (rabbit). Then the cells subjected to goat anti mouse or goat anti rabbit fluorescence antibody. DAPI was used for nucleus staining.

In order to express CD40L/GM-CSF in cis along with HTNV antigens, we developed the VLPs by inserting the membrane bound form of a murine CD40L/GM-CSF gene downstream of the HTNV M gene of our plasmid that expresses HTNV GP. We verified the plasmid by nucleic acid electrophoresis and bands can be seen at 780 bp, and 530 bp (Supplementary Figure S1A), 1.3 kb, 3.4 kb (Supplementary Figure S1B), corresponding with the length of S, M, CD40L and GM-CSF gene. The plasmids were further verified by DNA sequencing (Supplementary Figure S2). Indirect immune fluorescence analyses showed that the HTNV GP and CD40L/GM-CSF were expressed on the cell membrane after transfection (Figure 1). These results showed that CD40L/GM-CSF and HTNV GP could be expressed by co-transfection of plasmid pCI-S and pCI-M-CD40L, pCI-S and pCI-M-GM-CSF, which are used in further study.

### The morphology and protein composition of the VLPs is verified

**Figure 2:**
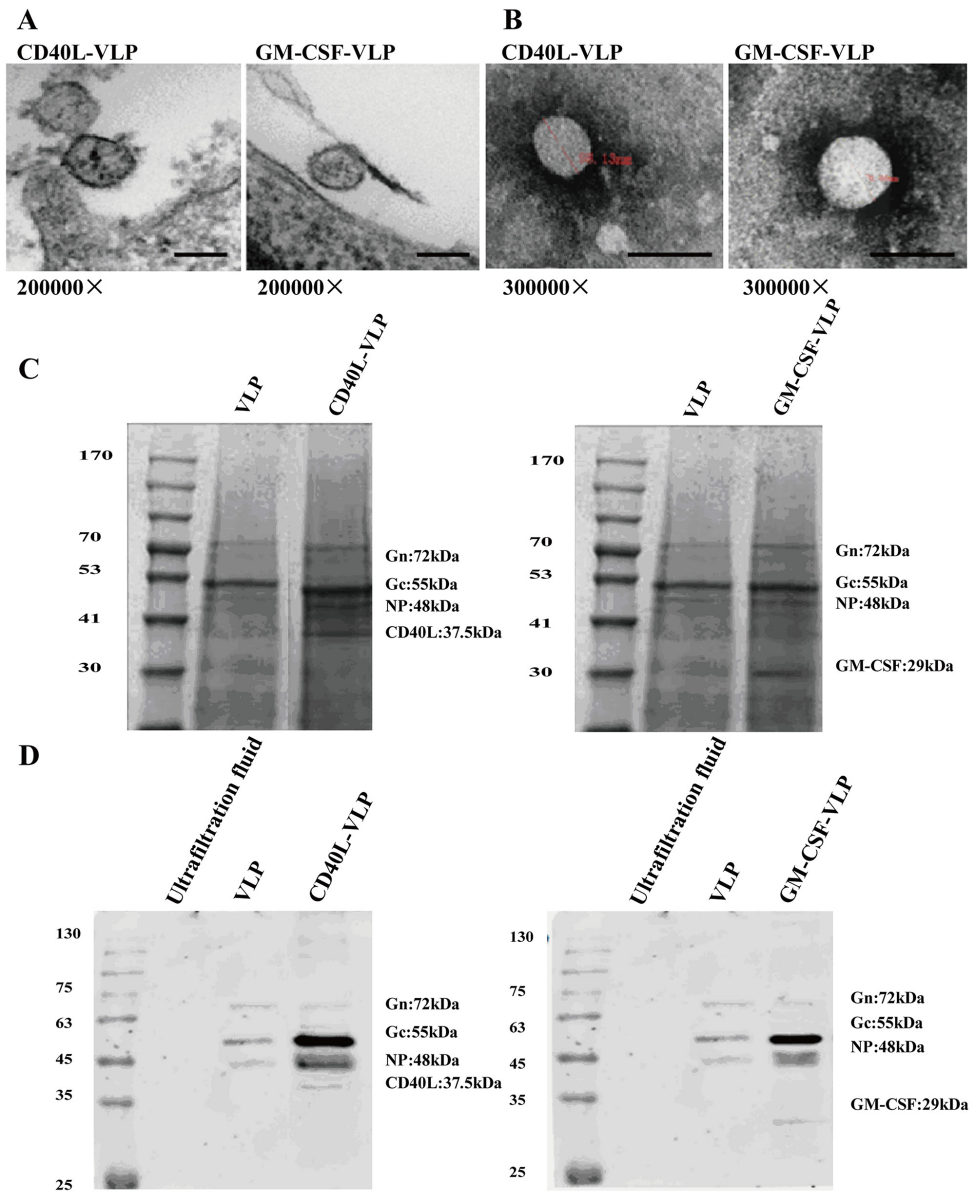
Verification of the morphology and protein composition of VLPs **A.** *Dhfr* deficient cells co-transfected with pCI-S and pCI-M-CD40L, pCI-S and pCI-M-GM-CSF were harvested and fixed with wax and sliced for electron microscopy. VLPs were observed besides cells ranged 50-100 nm in diameter. Each bar at the bottom indicates 100 nm. **B.** Purified VLPs were dropped onto bronze nets and stained with phosphoric acid, and then subjected to electron microscopy. VLPs ranged 80-120 nm in diameter were observed. Each bar at the bottom indicates 100 nm. **C.** Purified HTNV VLP, CD40L-VLP and GM-CSF-VLP were subjected to SDS-PAGE under 160 V for 45 min, and then stain with Coomassie blue. Bands locate at 70 kDa (Gn), 55 kDa (Gc), 48 kDa (NP), 37.5 kDa (CD40L) and 29 kDa (GM-CSF) can be observed. **D.** Purified HTNV VLP, CD40L-VLP and GM-CSF-VLP were subjected to SDS-PAGE under the same condition with (B), and then transferred to PVDF membrane. The membrane was then incubated with a mixture of GP monoclonal antibody and CD40L/GM-CSF polyclonal antibody. Corresponding secondary antibodies were incubated and scanned with a infrared imager.

We produced HTNV VLPs in *dhfr*^−^ CHO cells by co-transfected with pCI-S and pCI-M, pCI-S and pCI-M-CD40L, pCI-S and pCI-M-GM-CSF. Purified HTNV VLPs exhibited a consistent size of approximately 100 nm under transmission electron microscopy, in addition, HTNV VLPs were spherical and pleomorphic both in transfected cells (Figure 2A) and culture supernatants (Figure 2B). Purified HTNV VLPs proteins were confirmed using SDS-PAGE, Coomassie Blue-stained gels and Western blots. The expected molecular weights of Gn, Gc, NP and CD40L/GM-CSF (70, 55, 48 and 37.5/29kDa, respectively) were observed in purified HTNV VLPs (Figure 2C, 2D). All the VLPs got desired composition both in morphology and protein composition after purification.

### CD40L/GM-CSF HTNV GP were coexpressed

**Figure 3:**
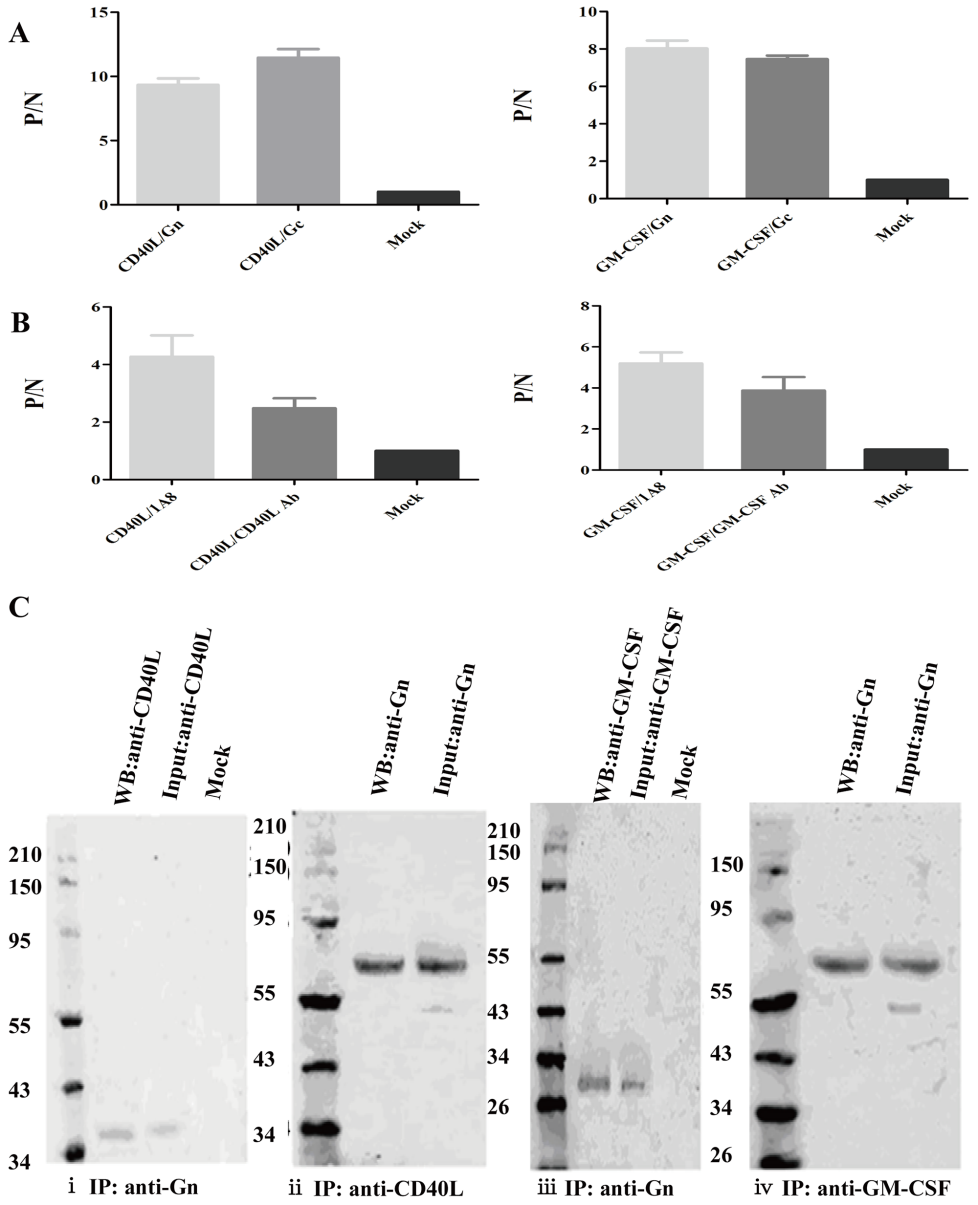
Verification of the co-localization of HTNV structural protein and CD40L/GM-CSF **A.** ELISA assay of purified VLPs. The 96-well plates were coated with Gn or Gc antibodies and detected with CD40L or GM-CSF antibody, the error bar is shown on the top of each bar. **B.** ELISA assay of purified VLPs. The 96-well plates were coated with NP specific antibody (1A8) or CD40L/GM-CSF specific antibodies (CD40L Ab/GM-CSF Ab) and detected with antibodies to CD40L or GM-CSF, the error bar is shown on the top of each bar. **C.** Western blot of samples of immunoprecipitation. CD40L (i) or GM-CSF (iii) was pulled down by Gn specific antibody and band of CD40L and GM-CSF can be seen at 37.5 kDa and 29kDa on PVDF membrane. Gn can also be pulled down by CD40L or GM-CSF antibodies, band of Gn can be seen at 70 kDa (ii iv).

We probed VLPs by indirect ELISA analysis, and CD40L/GM-CSF can be detected by Gn or Gc capture antibodies (Figure 3A). Similar results were obtained with NP specific monoclonal antibodies as capture antibodies (Figure 3B). The positive/negative (P/N) value of CD40L-VLP group and GM-CSF group is significantly higher than the control group (p<0.001). Immunoprecipitation (IP) showed similar results that CD40L/GM-CSF can be pulled down with Gn monoclonal antibodies (Figure 3C). These results suggest that the VLPs we constructed had desired protein colocalization, and were structurally intact.

### CD40L-VLP and GM-CSF-VLP promote differentiation of bone marrow-derived macrophages and dendritic cells *in vitro*

**Figure 4:**
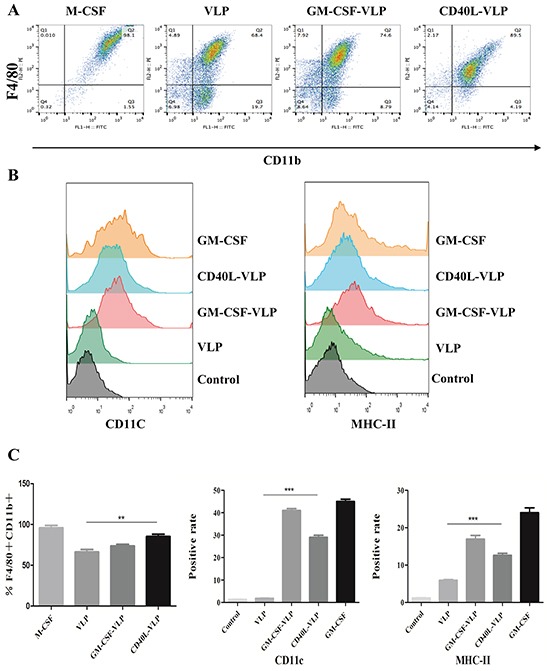
flow cytometry assay of macrophages and DCs stimulated by CD40L-VLP or GM-CSF-VLP Bone marrow cells were separated from healthy C57BL/6 mice and stimulated with CD40L-VLP, GM-CSF-VLP, undecorated VLP (VLP) and a M-CSF/GM-CSF positive control. **A.** The percentage of CD11b^+^ F4/80^+^ cell population of bone marrow cells stimulated by M-CSF, undecorated VLP (VLP), GM-CSF-VLP and CD40L-VLP respectively. **B.** The positive rate of CD11C and MHC-II of bone marrow cells stimulated with GM-CS, CD40L-VLP, GM-CSF-VLP or VLP. **C.** Quantitative analysis for (A) and (B)

We separated bone marrow cells from healthy C57BL/6 mice and stimulated with CD40L-VLP, GM-CSF-VLP, undecorated VLP (VLP) and M-CSF/GM-CSF as positive controls. As shown in Figure 4, the cell population of F4/80^+^CD11b^+^ in the GM-CSF-VLP and CD40L-VLP group (89.5% and 74.6%) are significantly higher than in the VLP group (68.4%) (p=0.0065) (Figure 4A), which indicated the differentiation towards bone marrow derived macrophages (BMDM). Meanwhile, the positive rate of bone marrow cells stimulated by CD40L/GM-CSF decorated VLPs showed significantly higher up-regulation of CD11c (29.0% and 41.0%) and MHC-II (12.6% and 17.0%) than the VLP group (1.9% for CD11b and 5.9% for MHC-II) (p<0.0001) (Figure 4B) which are markers for DCs (Figure 4C). These results indicate that CD40L/GM-CSF decorated HTNV VLPs have the potential of promoting the differentiation of bone marrow cells towards macrophages and DCs.

### CD40L-VLP and GM-CSF-VLP induced higher virus antigen specific antibodies and neutralizing antibodies as well as switching amongst antibody subtypes

**Figure 5:**
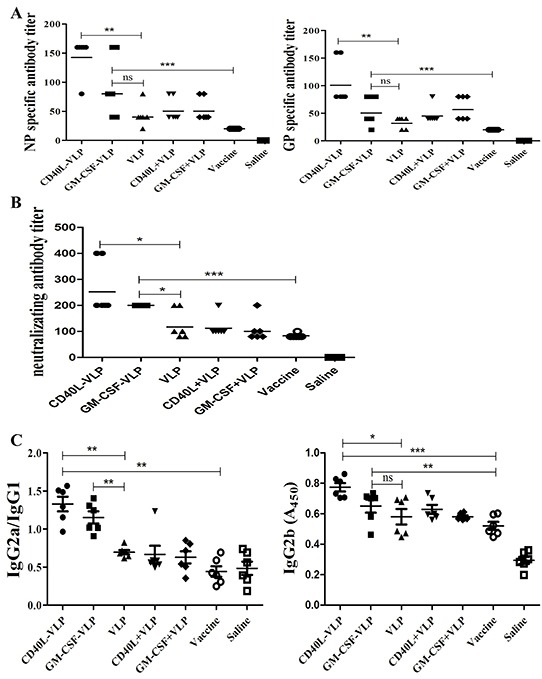
Detection of HTNV specific antibodies and antibody subtypes 7 days after the last immunization, sera from the mice were collected and subjected to **A.** HTNV GP or NP specific antibody detection by ELISA. 96-wells plates were coated with full length Gn and Gc or NP, a HRP conjugated goat anti mouse IgG antibody was used for detection of specific antibody in the sera. **B.** Sera from immunized mice were serial diluted and incubated with infectious HTNV before added to Vero E6 cells. 10 days later the cells were subjected to freeze/thaw cycles and the supernatant was collected for detection of HTNV virions by ELISA. The Geometry mean titer of neutralizing antibody titer was calculated. **C.** Sera from each group of mice were tested for immunoglobulin subtypes by ELISA. HRP conjugated goat anti mouse IgG1, IgG2a and IgG2b antibodies were used for detection. The relative values of absorbance at 450 nm of IgG2a to IgG1 (IgG2a/IgG1) was calculated, the values of absorbance at 450 nm of IgG2b was directly shown.

Purified recombinant VLPs antigens were tested for the ability to induce specific antibodies against each HTNV NP antigen by ELISA. Sera were obtained from each immunized mice and tested to assess HTNV antigen specific antibodies. NP or GP specific antibody titers were higher after immunization with CD40L-VLP compared to other VLP groups and vaccine group (p=0.0022 for NP and p=0.0015 for GP). However, GM-CSF-VLP group did not have advantage over undecorated VLP groups (p=0.1038 for NP and p=0.0928 for GP), but got advantage over vaccine and saline controls (p=0.0003 for NP and p=0.0004 for GP) (Figure 5A). We did not detect specific antibodies in saline control group. The Geometry mean titer of each group was listed (Table 1).

**Table 1:** Geometry mean titer of NP or GP specific antibody in the sera of immunized mice

Groups	NP specific (GMT)	GP specific (GMT)
CD40L-VLP	142.5	100.8
GM-CSF-VLP	80	50.4
VLP	40	31.7
CD40L+VLP	50.4	44.9
GM-CSF+VLP	50.4	56.6
Vaccine	20	20
Saline	--	--

NP or GP specific antibody titers obtained from ELISA were calculated for geometry mean titer. **NP specific** stands for HTNV NP specific antibody, **GP specific** stands for HTNV GP specific antibody. CD40L-VLP or GM-CSF-VLP stand for HTNV VLP decorated with CD40L or GM-CSF, CD40L+VLP or GM-CSF+VLP stand for CD40L or GM-CSF co-immunize with HTNV VLP.

Neutralizing antibody responses to HTNV were determined by ELISA analysis of the sera from immunized mice in order to evaluate the effective humoral immune response induced by the VLPs. There is no significant difference amongst VLP group and VLP co-immunized with CD40L/GM-CSF (p=0.2567). Compared with vaccine group and saline control, the geometric mean for the neutralizing antibody titer of the CD40L-VLP/GM-CSF-VLP were significantly higher (p=0.0003 for CD40L-VLP and p=0.0002 for GM-CSF-VLP) (Figure 5B). The mean for neutralizing antibody titer of the CD40L-VLP group and GM-CSF-VLP group was 251.9 and 200.0, which was higher than that of the undecorated VLP group (p=0.0113 for CD40L-VLP and p=0.0185 for GM-CSF-VLP). Sera from CD40L or GM-CSF immunized simultaneously with undecorated HTNV VLP mice showed similar neutralizing antibody titer with undecorated VLP group. These results suggested that immunization with recombinant VLPs can induced stronger neutralizing antibody responses than immunization with monovalent VLPs. The Geometry mean titer of each group was listed (Table 2).

**Table 2:** Geometry mean titer of neutralizing antibody in the sera of mice

Groups	Neutralizing antibody (GMT)
CD40L-VLP	251.9
GM-CSF-VLP	200
VLP	116.9
CD40L+VLP	100
GM-CSF+VLP	100
Vaccine	80
Saline	--

Neutralizing antibody titers obtained from ELISA were calculated for geometry mean titer. CD40L-VLP or GM-CSF-VLP stand for HTNV VLP decorated with CD40L or GM-CSF, CD40L+VLP or GM-CSF+VLP stand for CD40L or GM-CSF co-immunize with HTNV VLP.

Sera from immunized mice were tested for HTNV NP specific antibody subtypes by sandwich ELISA. We detected IgG1, IgG2a, IgG2b, IgA and IgM. The ratio of IgG2a/IgG1 is an indicator of humoral protective effect after viral infection. Sera from mice, which were immunized with CD40L-VLP or GM-CSF-VLP showed higher IgG2a compared to undecorated VLPs (p=0.0031 for CD40L-VLP and p=0.0033 for GM-CSF-VLP), however sera from vaccine group and PBS control showed higher IgG1. Though there were differences in IgG2b, the overall level of this subtype is low (Figure 5C). We also tested levels of IgA and IgM from sera of immunized mice, but there were no significant difference amongst VLP and vaccine group (p=0.3835 for IgA and p=0.4339) (Supplementary Figure S3). These results indicate that CD40L-VLP and GM-CSF-VLP may be able to switch immunoglobulin into specific subtypes.

### CD40L-VLP and GM-CSF-VLP promote anti-viral cytokines release and CTL response

**Figure 6:**
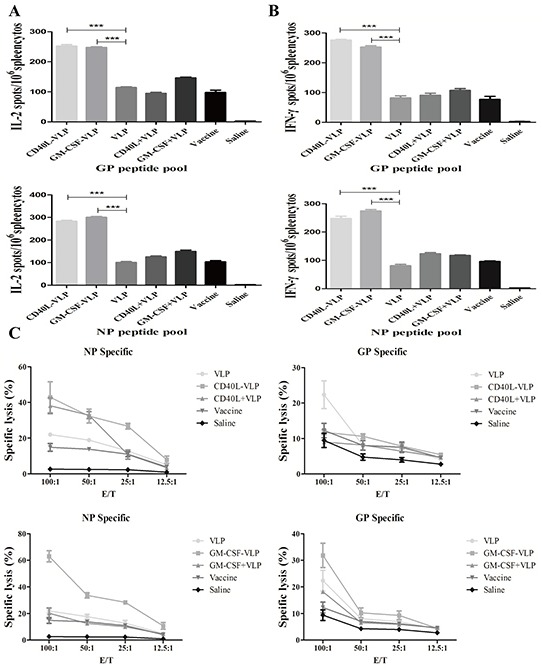
ELISPOT and CTL assay of splenocytes from immunized mice 7 days after the last immunization, splenocytes of the mice were isolated and stimulated with NP or GP peptide pool, each pool contain peptides of 15 amino acid residues with 8 overlaps. The splenocytes were tested for secretion of **A.** IL-2 through spot formation. **B.** Splenocytes from immunized mice were stimulated with the same peptide pool along with IFN-γ.**C.** HTNV infected macrophages from healthy mice were used as target cells. A serial of numbers of stimulated splenocytes were incubated with target cells for 6 h before the LDH release was assessed.

Spleen cells were isolated on day 34 (7 days after the last immunization) to assess the cellular immune response to the recombinant HTNV VLP. IFN-γ, IL-2, IL-4 and IL-10 cytokine were analyzed by ELISPOT, following stimulation with HTNV GP or NP peptides. There was no significant difference in levels of IFN-γ and IL-2 from undecorated VLP groups compared to inactivated HTNV virion vaccine group (p>0.05). The number of splenocytes secreting IFN-γ and IL-2 were higher in the CD40L or GM-CSF decorated VLP group compared to the vaccine group (Figure 6A, 6B). The overall amount of IL-10 and IL-4 secreting cells were small in all groups, however greater than the PBS control group; there was no significant difference in all VLP groups and vaccine group (Supplementary Figure S4). These results indicate that CD40L-VLP and GM-CSF-VLP may direct immune response towards Th1 type.

Macrophages could be infected by HTNV, which were used as target cells by immune fluorescence assay (Supplementary Figure S5), HTNV antigen can be observed on the membrane of macrophages and more than 90% of them were infected. Percentage of specific lysis of target cells was calculated and GM-CSF-VLP showed highest lysis activity stimulated by NP peptide. CD40L-VLP showed relatively moderate lysis activity. CD40L-VLP group showed significant difference compared to VLP groups under 100:1, 50:1 and 25:1 E/T (p<0.001, p<0.01 and p<0.01), when stimulated by NP peptide pool, but had no advantage over CD40L co-immunization group (p>0.05). When stimulated by GP peptide pool, CD40L-VLP showed advantage over VLP groups only under 100:1 E/T (p<0.0001). Under 100:1 E/T, GM-CSF-VLP group showed significant difference either compared with VLP groups (p<0.001) or with vaccine/saline control groups (p<0.001) when stimulated by GP peptide pool. When stimulated by NP peptide pool, GM-CSF-VLP showed advantage over any other group under 100:1, 50:1 and 25:1 E/T (p<0.001, p<0.001 and p<0.001) (Figure 6C). These results indicated that cellular immune response could be effectively induced by CD40L-VLP and GM-CSF-VLP, though CD40L-VLP induction was relatively moderate.

### Protection of mice from viral challenge

**Figure 7:**
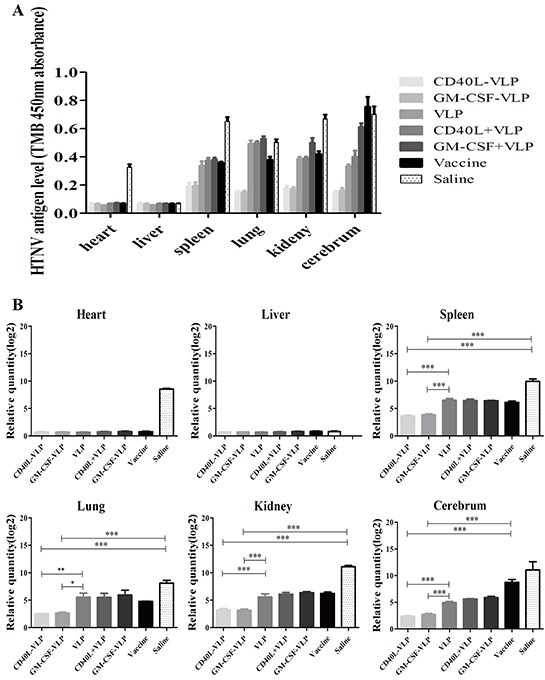
Detection of viral load in main organs after virus challenge 3 days after virus challenge of immunized mice, primary organs of all groups of mice were collected and grinded for HTNV specific antigen detection. **A.** The samples were subjected to freeze/thaw cycles and the supernatant were collected for ELISA. The level of NP protein was shown on the graph. **B.** Total RNA was extracted and reverse transcripted to cDNA. We used a S segment specific primer for detection of HTNV specific sequence, the relative quantity is show on the graph.

We tested the protection ability provided by VLPs by challenging immunized mice with infectious HTNV. Three days after viral challenge, the viral load of heart, liver, spleen, lung, kidney and cerebrum were tested by sandwich ELISA as well as qRT-PCR. We detected small amount of viral antigen (Figure 7A) and RNA (Figure 7B) exist in liver from all groups, however the amount of virus in spleen, lung, kidney and cerebrum were significantly lower in CD40L-VLP (p<0.0001 for heart, p<0.01 for spleen, p<0.001 for lung, p=0.001 for kidney and p=0.001 for cerebrum) and GM-CSF-VLP (p<0.0001 for heart, p<0.01 for spleen, p<0.001 for lung, p<0.001 for kidney and p<0.001 for cerebrum) groups. PBS control group showed highest amount of viral load. These results indicate that immunization of the recombinant VLPs can provide protection against HTNV challenge by reducing viral load.

## DISCUSSION

VLPs have similar structural and physicochemical features with origin virions, however they are non-infectious and possess advantages in terms of safety and manufacturing [23]. One of the important characteristics of VLPs is that they can elicit strong humoral and cellular immune responses against viruses. Recombinant Hantaan virus VLPs have been shown to be able to be efficiently produced by expression of M segment, either with or without nuclear proteins [9], but VLPs with full components may elicit stronger immune response. Our previous study demonstrated that the reconstructed pCI-neo plasmid can be used in protein expression by CHO cells (data unpublished). In this study, the reconstructed pCI-neo was applied to recombinant VLP production and the immunogenicity and protective effect of the VLPs were evaluated.

To ensure the expression of VLPs, we used a *dhfr* gene deficient CHO cell line, which cannot survive in medium without hypoxanthine and thymine (HT) supplement. The pCI-S-neo and pCI-M-CD40L/GM-CSF plasmid contain two parts of *dhfr* gene, only when the two plasmids are correctly transfected can the deficient cells survive in medium without HT. The *dhfr* deficient CHO cells are sensitive to G418, but pCI-S-neo can provide G418 resistance, that is to say, correctly transfected cells can survive in medium containing G418. We also applied MTX in culture medium, which can suppress the expression of *dhfr* gene; it must be amplified to keep cells alive. CD40L/GM-CSF was located downstream of *dhfr*, the amplification can spur the expression of CD40L/GM-CSF. These measurements can ensure the correct expression of all components of the VLPs we needed. *In vitro* stimulation with CD40L/GM-CSF decorated VLPs was able to promote bone marrow cells towards DCs and macrophage lineage, which indicate that VLPs had desired construction and basic function.

The analysis of humoral immune responses revealed that antigen specific IgG CD40L-VLP and GM-CSF-VLP induced were higher than that of undecorated VLPs, and even higher than HTNV vaccine used in clinical practice. We also found that antibody subtypes changed in sera from immunized mice, CD40L-VLP and GM-CSF-VLP had highest ratio of IgG2a/IgG1, revealing the anti-viral ability of antibodies is high *in vivo* [24, 25]. Neutralizing antibody assay further proved this observation. VLPs can not only improve antibody production, but also enhance cellular immunity. In our study, anti-viral cellular immune responses were assessed by the number of VLP-stimulated spleen cells that release IFN-γ, IL-2, IL-4 and IL-10. The number of IFN-γ and IL-2 secreting splenocytes was significantly higher in mice that received CD40L or GM-CSF decorated VLPs compared to vaccine group and PBS controls. However, neither IL-4 nor IL-10 secreting splenocytes were significantly higher in all VLP groups. IFN-γ is a type of Th1 cytokine, which has an anti-viral effect, we can infer that the immunization of VLP may alter the tendency of immune response to Th1 [26], the low level of IL-4 and IL-10 secretion proved this hypothesis [27]. However, many other cytokine/chemokines are considered to be correlated with Hantaan virus related disease, including IL-2, IL-6, IL-8, IL-10, IFN-γ, TNF, and the intensity of platelet β3 integrin [28–34]. Results of the current study are not enough to evaluate Th1 or Th2 immune responses produced by CD40L or GM-CSF decorated VLPs or the corresponding dynamic variation. Further studies of T-cell responses are needed to understand the immune status of mice immunized with the recombinant HTNV-VLP.

The protective efficacy of the VLPs was tested by viral challenge of immunized mice. The results showed that immunization with either CD40L-VLP or GM-CSF-VLP conferred the most protection, against HTNV challenge, which has the least viral load in important organs. However histopathological analysis show little difference in numbers of lymphocytes except in spleen. This is probably because HTNV can only infect adult mice causing no pathological events and we intend to use suckling mouse to assess the protection effect of the VLPs in our further study.

In conclusion, the construction of a recombinant HNTV VLP vaccine candidate, based on *dhfr* deficient CHO cells and the immune effect of the VLPs is described. We successfully produced VLPs with desired protein components and construction. The CD40L or GM-CSF decorated VLP induced stronger humoral immune responses against HTNV as well as cellular immunity. Moreover, protective efficacy in mice was better with the CD40L or GM-CSF decorated VLP than with the vaccine. Further study is needed to adjust the productivity of the VLP producing *dhfr*^−^ CHO cells and to clarify the protective efficacy of that is induced.

## MATERIALS AND METHODS

### Cells and viruses

Dihydrofolatereductase deficient CHO cells (*dhfr*^−^ cells, purchased from Culture Collection of Chinese Academy of Science, Shanghai) were maintained in 37°C with 5% CO_2_ in ISCOVE's modified DMEM (IMDM) (Gibco) supplemented with 10% fetal bovine serum (Gibco), 2% HT mix (Sigma), and 100nM methotrexate (MTX) (Sigma). Mouse macrophage cells and bone marrow cells were separated from healthy C57BL/6 mice and similarly cultured in DMEM supplemented with 10% FBS. HTNV strain 76118 was kept in our lab, and all related experiments were performed in a biosafety level 2 (BSL-2) setting.

### Construction of CD40L or GM-CSF anchored VLP expressing plasmid

Genes encoding HTNV M, HTNV S segments were cloned into pCI-neo plasmid, which was provided by professor Yan Yan. We named the recombinant plasmid as pCI-S and pCI-M. pCI-M was digested with StuI and BstBI and then mouse CD40L gene was cloned into the plasmid. Mouse GM-CSF-GPI gene was similarly cloned into pCI-M using StuI and BstXI. The plasmids containing CD40L and GM-CSF gene were named as pCI-M-CD40L and pCI-M-GM-CSF respectively. Nucleic acid electrophoresis and DNA sequencing verified the construction of the four plasmids.

Indirect fluorescence assays (IFAs) were performed to assess the expression of GP, CD40L and GM-CSF. *Dhfr*^−^ cells were cotransfected with pCI-S and pCI-M, pCI-S and pCI-M-CD40L, pCI-S and pCI-M-GM-CSF. The cells were cultured at 37°C for 48h. Supernatants were discarded, and the cells were fixed with 0.4% polysorbate at room temperature for 15 min. Cells were then washed in PBS (0.01mol/L) and treated with 0.5% Triton-X100 at room temperature for 15 min. After washing with PBS, mouse anti HTNV Gc monoclonal antibody and rabbit anti CD40L or GM-CSF polyclonal antibodies were incubated with fixed cells at 37°C for 1h. Cells were then washed with PBS and incubated with FITC-conjugated goat anti-mouse and Cy3-conjugated goat anti-rabbit secondary antibody at 37°C for 1h. DAPI was used for nuclear dye. Finally, the cells were washed with PBS and observed using fluorescence microscopy.

### VLP production and purification

*Dhfr*^−^ cells were cotransfected with pCI-S and pCI-M, pCI-S and pCI-M-CD40L, pCI-S and pCI-M-GM-CSF respectively. The cells were cultured for 24h after transfection, and then cells were carefully washed with DPBS before replacing culture medium with serum-free CHO medium (Hyclone). Supernatants were collected 72h after transfection. Culture supernatants containing VLPs were harvested and clarified by low-speed centrifugation at 1000rpm for 10 min at 4°C followed by ultracentrifugation at 30,000rpm for 60 min to pellet. Sedimented particles were suspended in phosphate-buffered saline (PBS) at 4°C.

### Verification of VLPs

Characterization of HTNV VLPs GP, NP and CD40L or GM-CSF was performed using a sodium dodecyl sulfate (SDS) 10% polyacrylamide gel; Coomassie stain and Western blot analysis. Total VLP protein amounts of 10μg, were loaded into the gel and transferred onto a PVDF membrane using a Mini Trans-Blot (Bio-Rad, CA). Membranes were blocked using 5% BSA at room temperature for 1h and then incubated with HTNV Gc monoclonal antibody and CD40L or GM-CSF polyclonal antibodies overnight at 4°C. Membranes were washed with PBS containing 0.5% tween-20 (PBST), and then incubated with infrared conjugated antibody and proteins were visualized using an infrared imaging system.

Coexpression of HTNV GP and CD40L or GM-CSF was examined by ELISA. We coated a 96-well plate with Gn or Gc monoclonal antibodies at 4°C overnight, the plate was washed with PBST, then samples containing CD40L-VLP and GM-CSF-VLP were addes into the plate and incubated at 37°C for 1 h. Anti CD40L or GM-CSF antibody was added into the plate and incubated at 37°C after washing with PBST. The uncombined antibodies were washed away and HRP conjugated secondary antibodies were added. After incubation at 37°C for 1h, TMB solution was added and 450nm absorbance was detected. The coexpression was further tested by immunoprecipitation. Purified CD40L-VLP and GM-CSF-VLP were incubated with CD40L/GM-CSF antibody or Gn antibody at 4°C overnight. Then we used Protein G beads to capture the antibodies. The samples were boiled with SDS and the beads were discarded before they were subjected to Western blot.

### Electron microscopy

*Dhfr*^−^ CHO cells cotransfected with pCI-S and pCI-M, pCI-S and pCI-M-CD40L, pCI-S and pCI-M-GM-CSF expressing HTNV-VLP, CD40L-VLP and GM-CSF-VLP respectively, were fixed with 1% phosphotungstic acid for 1-2 min to examine the budding of VLPs at 200,000×. Supernatant was dropped onto a piece of bronze net and excess stain was wicked away. The samples were air dried for 1-3 min. VLPs were observed using a H8100 transmission electron microscope at a magnification of 300,000X.

### Determination of *in vitro* activity of VLPs

Bone marrow was flushed from femurs and tibias from healthy C58BL/6 mice. A total of 1× 10^6^/well nucleated cells were added to 6-well tissue culture plates and cultured in 1 mL of RPMI1640 medium containing 10% FCS. Then the VLPs were added to observe differentiation toward macrophage/DCs lineage that was compared with M-CSF or GM-CSF positive control. After 6 days in culture, contaminating non-adherent cells are eliminated and adherent cells are harvested for BMDM assays. Cells were cultured for 6 d and then harvested for flow cytometry. Population of macrophages is determined by staining with F4/80 and CD11b antibodies, DCs were determined with CD11c and MHC-II antibodies.

### Flow cytometry

FACS analysis were performed with routine protocols using the FACS Calibur flow cytometer (BD Immunocytometry Systems), antibodies used for mice spleen lymphocyte staining are as following: CD3-Percp cy5.5, CD4-FITC, APC, CD8-Percp cy5.5, PE, CD19-FITC, B220-PE, CD69-FITC, CD80-FITC, CD86-APC, CD40-PE (Biolegend) and bone marrow staining: F4/80-PE, CD11b-FITC, MHC-II-PerCP cy5.5 (Biolegend). FACS buffer; 1 × PBS (Gibco) + 2% FBS (Sigma). All data were analyzed using FlowJo (Tree Star, Inc.).

### Mice immunization

Experiments with mice were conducted in compliance with a protocol approved by the Institutional Animal Care and Use Committee based on the Ethical Principles in Animal Experimentation. Specific pathogen-free female C57BL/6 mice aged four to five weeks, were divided into seven groups according to immunogen, each group contains 12 mice. Animals were inoculated intraperitoneally (i.p.) with CD40L-VLP, GM-CSF-VLP, and undecorated HTNV-VLP respectively (100μg per dose and in 12 mice (n=12)). Same number of mice was inoculated intraperitoneally with HTNV-VLP and mouse CD40L (VLP+CD40L group, 100 μg HTNV-VLP and 25ng CD40L per dose), HTNV-VLP and mouse GM-CSF (VLP+GM-CSF group). HTNV vaccine (β-propiolactone inactive HTNV viron) was subjected to equal number of mice (100μl per dose). Equal amounts of PBS were used as controls (Table 3). Blood samples were collected on days 0, 14 and 28, via the tail vein, for measurement of serum IgG. Seven days after the last inoculation, 2/3 of the mice in each group were euthanized. The spleen of each mouse was removed and splenocytes were isolated in order to test cytokine profiles; in adiition serum was collected for further immunological analysis.

**Table 3:** Immunization dose for animal experiments

Groups	Doze
CD40L-VLP	100 μg/100 μl/mouse
GM-CSF-VLP	100 μg/100 μl/mouse
VLP	100 μg/100 μl/mouse
CD40L+VLP	100 μg/100 μl+25 ng CD40L/mouse
GM-CSF+VLP	100 μg/100 μl+25 ng GM-CSF/mouse
Vaccine	10 μl+90 μl PBS/mouse
Saline	100 μl/mouse

Doze of immunization for each mouse is listed in this table. CD40L-VLP or GM-CSF-VLP stand for HTNV VLP decorated with CD40L or GM-CSF, CD40L+VLP or GM-CSF+VLP stand for CD40L or GM-CSF co-immunize with HTNV VLP.

### Humoral immune response assay

Sera from each group of mice were assessed for immunoglobulin by ELISA. Full length HTNV NP or Gc (0.15μg) was coated to each well of 96-well plates and incubated at 4°C overnight. The sera were serially diluted with PBS from 1:20 to 1:1024 and add into NP/Gc coated 96-well plates. After incubation at 37°C for 1h, plates were washed with PBST and then diluted HRP conjugated goat anti mouse IgG antibody was added to each well. Subsequently plates were incubated at 37°C for 1h and then washed with PBST, then TMB solution was added and absorbance at 450 nm was recorded. The titer was determined in samples that had maximum dilution still showing positive result.

In order to distinguish the immunoglobulin subtypes, full length HTNV NP (0.15μg) was coated using similar method above. Sera from each group of mice were diluted and HRP conjugated IgG to mouse IgG1, IgG2a, IgG2b, IgA and IgM were applied. Absorbance at 450 nm was assessed to determine the amount of each immunoglobulin subtypes.

Because HTNV do not form plaque in Vero E6 cells, the neutralizing ability of antibodies was measured using a sandwich ELISA method. Briefly, Vero E6 cells were grown to 60% confluence in 96-well plates. Then plates were pre-incubated with serial dilutions of mouse serum (1:80, 1:100, 1:200,1:400, 1:800 and 1:1600) together with HTNV (1×10^−5^ TCID_50_) at 37°C for 1h. The virus-serum mixture was aspirated, added along with 0.2 ml overlay medium, and incubated at 37°C with 5% CO2 for 10 days. Plates were subjected to RT/−80°C freeze-thaw cycles 3 times and the supernatant was collected for ELISA. We used HTNV NP monoclonal antibody as capture antibody and HRP-1A8, which is an HRP conjugated HTNV NP monoclonal antibody, as detecting antibody. Neutralizing antibody titers are defined as the maximal serum dilution that 3 out of 4 duplicated wells do not show positive of HTNV NP.

### Cytokines ELISPOT assay

The cytokines, IFN-γ, IL-2, IL-4 and IL-10, from immunized mice were measured using ELISPOT kits (Mabtech), according to the manufacturer's instructions. Briefly, ELISPOT 96-well plates (supplied by the kit) were coated with 100 μl of anti-mouse IFN-γ, anti-mouse IL-2, anti-mouse IL-10, or anti-mouse IL-4 (5μg/ml in coating buffer). The plates were washed twice and blocked with blocking solution for 2h. A volume of 100μl freshly isolated splenocytes (1× 10^6^ cells) from the immunized mice were subsequently transferred to each well and stimulated with NP or Gn/Gc peptide pools at 37°C for 24 h. The cells were then washed away, and a secondary biotinylated anti-cytokine mAb was added to each well, followed by a streptavidin-HRP and AEC substrate solution system. Finally, the spots were counted using an ImmunoSpot^®^ Analyzer (Cellular Technology Ltd. USA)

### Cytotoxic assay of splenocyte

Macrophages separated from healthy mice were infected with 100 TCID_50_ HTNV at 37°C for 1h, excessive virus was then washed away and DMEM supplemented with 10% FBS was added to the cells. The macrophages were tested by immune fluorescence to assess the infection rate 2d later; the HTNV infected macrophages were used as target cell. Splenocytes were separated from immunized mice and stimulated with IL-2 as well as the same peptides as ELISPOT assay. U shaped 96-well plates were used; a volume of 100 μl target cells (1× 10^4^ cells) was added to each well. Then a serial of different numbers of splenocytes were applied to the wells (1× 10^6^ cells, 5× 10^5^ cells, 2.5× 10^5^ cells, 1.25× 10^5^ cells). The plates were incubated at 37°C for 6 h, and lactate dehydrogenase (LDH) released was assessed. The percentage of specific lysis was calculated using the following formula.

$$\text{Specific cell lysis}(\%)=\frac{\text{experiment group}-\text{LDH self release of target cells}-\text{LDH self release of splenocytes}}{\text{LDH max release of target cells}-\text{LDH self release of target cells}}$$

### Mice protection against viral challenge

Four mice from all 7 groups were injected intracerebrally with 100μl of infectious HTNV. Three days after injection, the mice were euthanized and tissues from heart, liver, spleen, lung, kidney and cerebrum were collected and divided into two halves. One half was weighted and triturated in PBS. The total protein was measured by BCA assay and all samples were diluted to 1mg/ml, then HTNV antigen was analyzed using the same ELISA method with neutralizing antibody assay.

Total RNA of the other half of tissue was extracted following manufacturer's instructions (Axygen), and reverse transcripted into cDNA using a reverse transcription kit (Takara). Copies of HTNV S segments were counted through RT-PCR analysis using a SYBR green RT-PCR kit (Takara), and mouse GAPDH was used as reference.

### Statistical analysis

Data were analyzed for statistical significance using GraphPad Prism 5 software. Percentage of flow cytometry was compared using one-way ANOVA, and data were expressed as the mean ± SEM. Statistical analysis of antibody titer was performed using Kruskal-Wallis test, and titers were expressed as the geometric mean. Comparison of antibody subtype level were also using Kruskal-Wallis test. Results of ELISPOT assay were compared by one-way ANOVA, and CTL assay was compared using two-way ANOVA. Protective efficacy was compared using one-way ANOVA. Data was presented as the mean ± SEM.

## SUPPLEMENTARY MATERIALS FIGURES


