# Supplementary material for: Construction and immunological characterization of CD40L or GM-CSF incorporated Hantaan virus like particle
Source: Oncotarget. 2016 Aug 17;7(39):63488–503. doi: 10.18632/oncotarget.11329 (PMC5325379; doi:10.18632/oncotarget.11329)
Supplement: Supplementary file 1 [file oncotarget-07-63488-s001.pdf]

Construction and immunological characterization of CD40L or GM-CSF incorporated hantaan virus like particle

SUPPLEMENTARY FIGURES

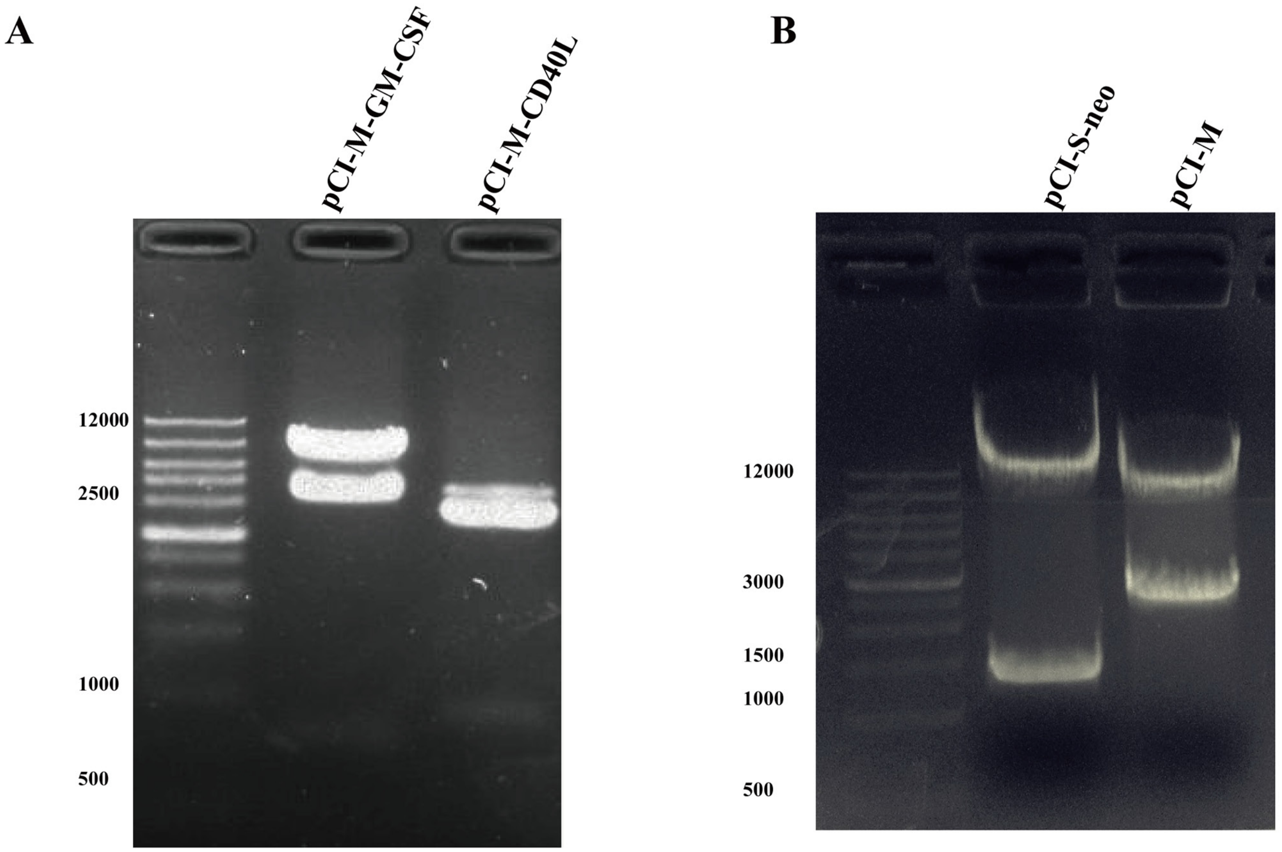

Supplementary Figure S1: Nucleic acid electrophoresis. pCI-M-CD40L and pCI-M-GM-CSF were digested with StuI and BstXI, pCI-S and pCI-M were digested with StuI and BstBI, and then subjected to nucleic acid electrophoresis. The bands locate at 530 bp (GM-CSF) and 780 bp (CD40L) **A.** 1.3 kb (S segment) and 3.4 kb (M segment) **B.**

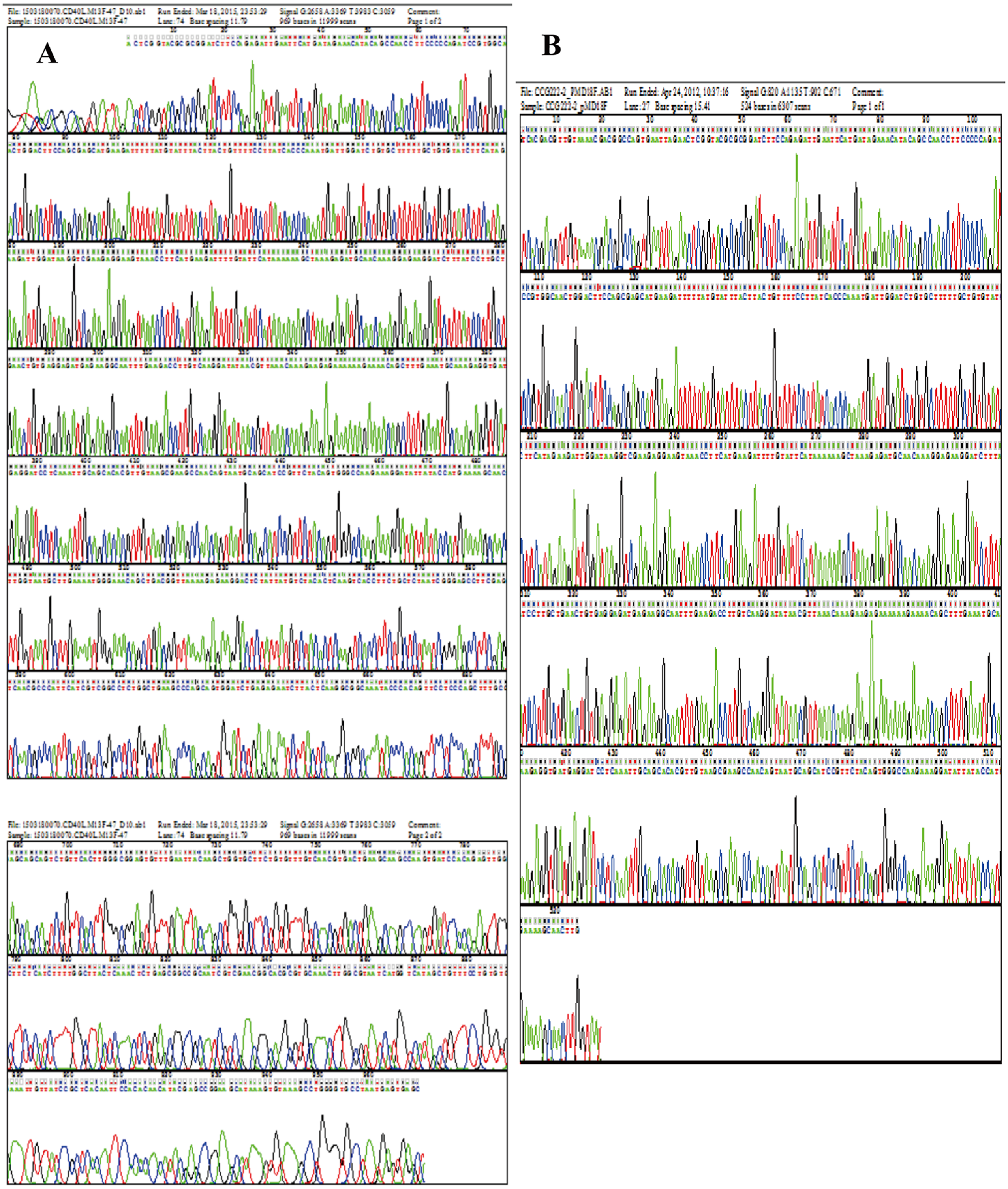

Supplementary Figure S2: DNA sequencing. pCI-M-CD40L and pCI-M-GM-CSF were digested and CD40L**A.**/GM-CSF**B.** gene were salvaged and subjected to DNA sequencing.

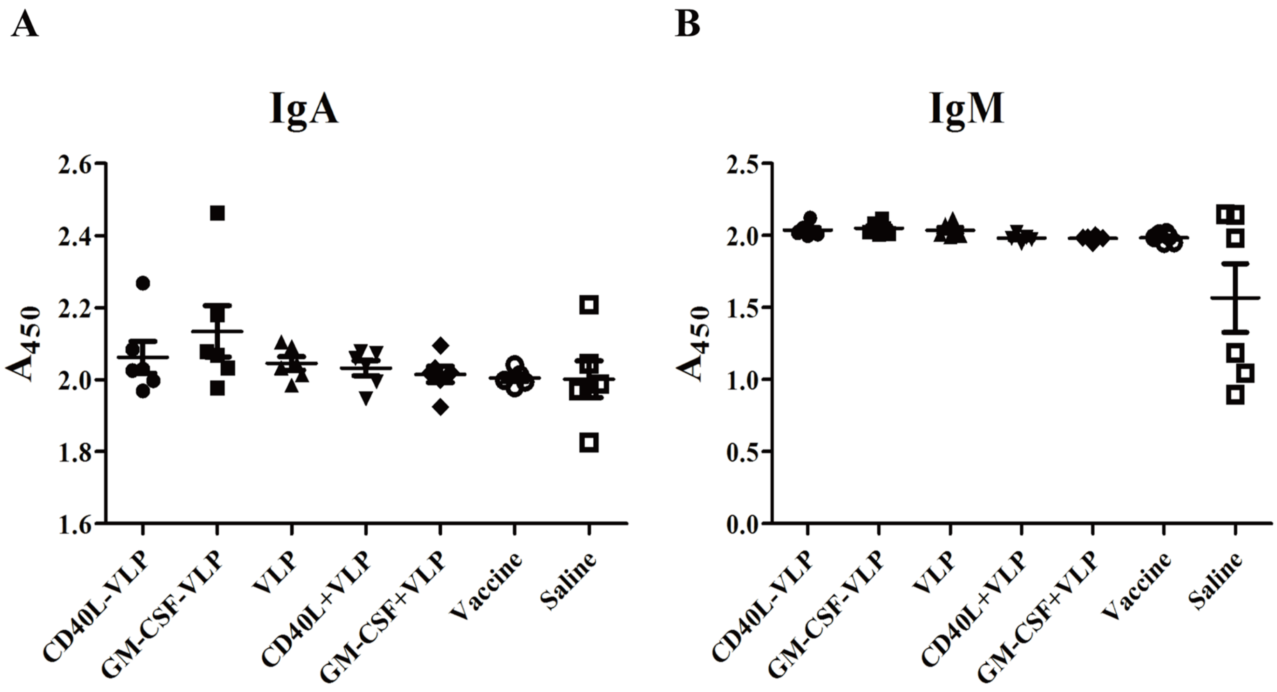

Supplementary Figure S3: IgA and IgM in sera from immunized mice. Sera from immunized mice were tested for antibody subtypes and by ELISA. HRP conjugated anti mouse IgA and IgM antibodies were used as detecting antibody. There was no significant difference between VLP groups and vaccine group ($p>0.1$) either for IgA **A.** or IgM **B.**

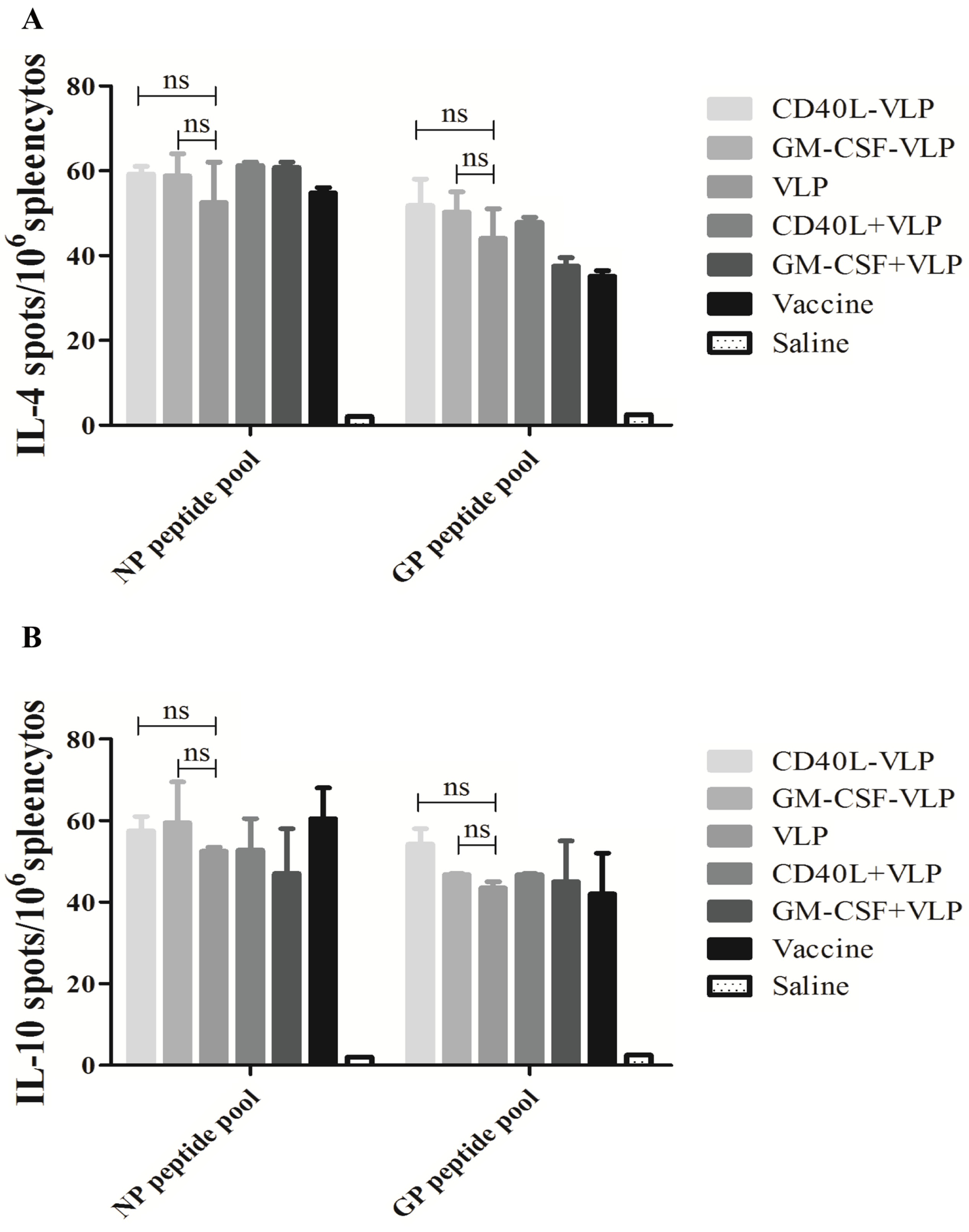

Supplementary Figure S4: IL-4 and IL-10. 7 days after the last immunization, splenocytes of each group were separated and tested for IL-4 **A.** and IL-10 **B.** secretion by ELISPOT. There is no significant difference between VLP groups and vaccine group ($p>0.1$). Almost no spots were observed in Saline control group.

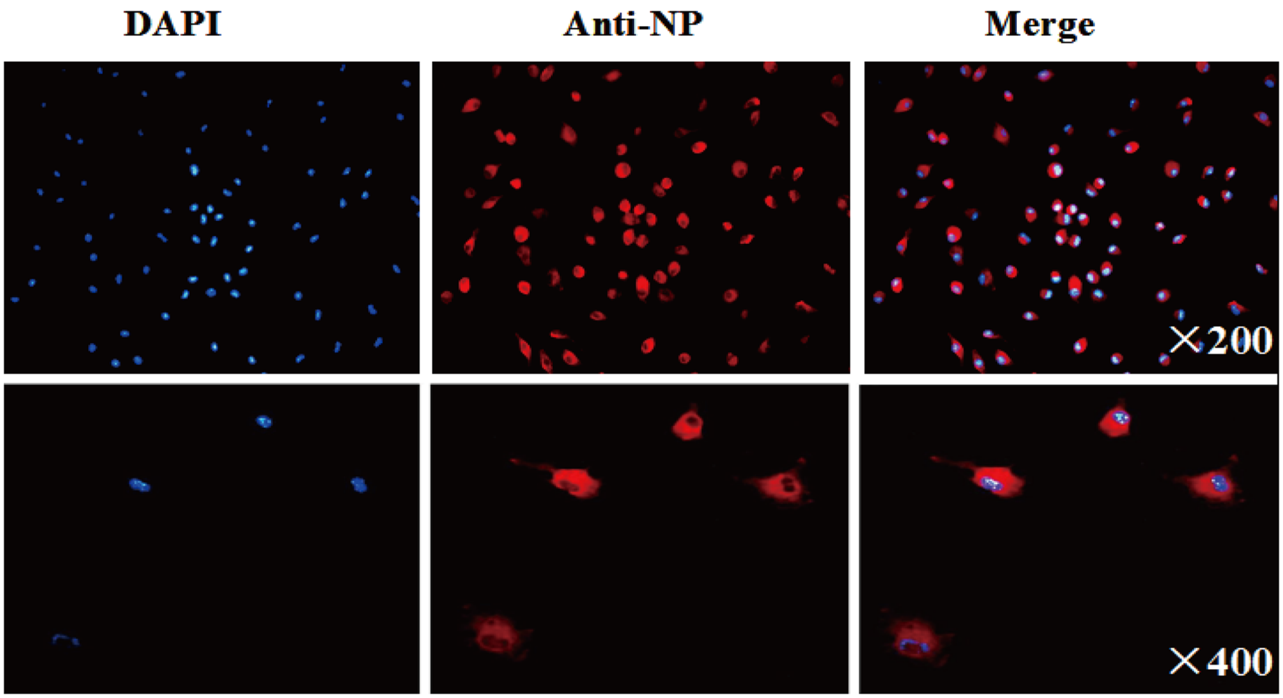

Supplementary Figure S5: Macrophages infected by HTNV. Macrophages were separated from healthy C57BL/6 mice and infected by HTNV. The antigen presenting ability was tested by detecting NP. We used a NP specific monoclonal antibody (mouse) and Cy3 conjugated goat anti mouse antibody for detection. Fluorescence microscopy showed intense NP antigen on and in macrophages.
